# Founder events and pre-glacial divergences shape the genetic structure of European Collembola species

**DOI:** 10.1186/s12862-016-0719-8

**Published:** 2016-07-16

**Authors:** Helge von Saltzwedel, Stefan Scheu, Ina Schaefer

**Affiliations:** Johann Friedrich Blumenbach Institute of Zoology and Anthropology, Georg August University Göttingen, Berliner Strasse 28, 37073 Göttingen, Germany

**Keywords:** Colonization, Springtail, Quaternary, Founder takes it all, Miocene divergence, Climate change, Genetic diversity, Parthenogenesis

## Abstract

**Background:**

Climate oscillations in the Cenozoic reduced species richness and genetic diversity of terrestrial and aquatic animals and plants in central and northern Europe. The most abundant arthropods in temperate soils are Collembola that live in almost any soil-related habitat. Extant species show little morphological variation to Eocene fossils, suggesting persistence of species in stable habitats for millions of years. Collembola are able to evade adverse climatic conditions by moving into deeper soil layers and are tolerant to frost and draught. If these adaptations sufficed for surviving glacial periods remains open and needs to be investigated in a phylogeographic context, i.e. investigating spatial structure on molecular level. We investigated the molecular variation of three common species of Collembola at a pan-European scale to identify glacial refuges and post-glacial colonization patterns with three genetic markers.

**Results:**

All genes revealed remarkable genetic structure between but not within populations, suggesting density dependent processes for establishment of populations (founder-takes-all principle), which is common for European animals and plants. In contrast to the post-glacial recolonization patterns of many aboveground organisms, divergence times of most geographic lineages indicate preservation of genetic structure since the Miocene.

**Conclusions:**

Collembola survived severe climatic changes including those during Quatenary glaciation and kept high genetic variance across Europe. Likely the buffering of temperature oscilliations in soil and the ability to evade adverse climatic conditions due to cold-tolerance and horizontal migration enabled Collembola to evade strong selective pressure of abiotic forces.

**Electronic supplementary material:**

The online version of this article (doi:10.1186/s12862-016-0719-8) contains supplementary material, which is available to authorized users.

## Background

Collembola are small wingless hexapods that have been among the first arthropods on land, oldest fossils date to the Early Devonian ~400 mya [1, 2]. Fossils from Baltic amber (55–35 mya) have been assigned to extant species [3–6], indicating persistence with little morphological modification over very long periods of time. They are worldwide distributed and are ubiquitous in leaf litter with more than 90 % of the individuals inhabiting the upper 10 cm of the soil [7]. They reach high local density in temperate forests (> 10^5^ ind./m^2^; [8]) and significantly contribute to decomposition processes, soil respiration and nutrient cycling [9, 10].

The above-and belowground food web is intimately linked [11] but also differs in many respects. Mobility of soil arthropods is limited due to the porous structure of soils. Further, abiotic constraints, such as temperature fluctuations and drought, are less severe in soil, and soil-living animals can evade adverse climatic conditions by moving deeper into the soil [12, 13]. Indeed, freezing avoidance but also frost and draught tolerance is widespread in soil invertebrates, including Collembola [14–17]. Consequently, Collembola are little affected by low temperature conditions and in temperate ecosystems they typically remain active during winter [18–20]. This suggests that Collembola in soil may have suffered less from Quaternary climate changes than animals above the ground.

Collembola form part of the decomposer system and predominantly feed on dead organic matter and associated microorganisms [21, 22], resources which likely were at least temporarily available during Quaternary glaciations of central Europe. Therefore, present day populations of central European Collembola may well descend from relict populations that survived glacial periods, rather than from south European populations that recolonized empty habitats when glaciers retreated northwards. Effects of the recurrent and severe abiotic disturbances during Quaternary ice-ages on the genetic structure of soil-living animals has been little investigated [23, 24], disregarding one of the most species-rich terrestrial animal communities.

Few studies investigated the genetic structure of Collembola in Europe and the existing studies focused on large surface-dwelling rather than soil-dwelling species [25–28]. Generally, there is very limited information on phylogeographic patterns of soil-living invertebrates across Europe. The only study available analyzing the genetic structure of soil-living arthropods on a wide geographic area investigated the oribatid mite species *Steganacarus magnus* [24]. In agreement with the above considerations the results suggest that *S. magnus* survived Quaternary glaciations in central Europe in cryptic refugia and indicates that phylogeographic patterns of aboveground animals cannot easily be transferred to those of the belowground system. Collembola resemble oribatid mites in various respects but typically are faster reproducing, more mobile and less frost tolerant [19, 29–33].

The ability to survive Quaternary glaciations and the recolonization potential from glacial refuges likely differs between Collembola and oribatid mites. We investigated the genetic structure of three species of Collembola that are widely distributed and common in the northern hemisphere across thirteen European countries, including potential refuge areas south of the Alps. *Ceratophysella denticulata* (Bagnall, 1941) (Hypogasturidae) and *Folsomia quadrioculata* (Tullberg, 1871) (Isotomidae) are large species (up to 2.5 mm) that live in the uppermost soil layer (hemiedaphic) and reproduce sexually. In contrast, the third species, *Isotomiella minor* (Schaeffer, 1896) (Isotomidae), is small (up to 1.3 mm), lives deeper in soil (euedaphic) and reproduces via parthenogenesis. The investigated species are generally described as palearctic or holarctic in their distribution ranges, only *I. minor* is considered cosmopolitan, but its taxonomic status needs further attention [34–36]. In fact, all three species are more widely distributed but the part of introduced and indigenous populations in other regions of the world remains uncertain [37]. *Ceratophysella denticulata* feeds on bacteria and fungi but presumably also on other soil animals such as nematodes [21, 38]. While *C. denticulata* functions as secondary decomposer, *F. quadrioculata* is a typical primary decomposer feeding predominantly on litter [21]. In contrast, *I. minor* presumably mainly feeds on bacteria [39, 40]. Due to its parthenogenetic mode of reproduction, *I. minor* likely recovers more quickly from disturbances and colonizes new habitats faster than sexual species [32]. Species of Hypogasturidae and Isotomidae have been found in Baltic amber (55–35 mya) [3–5, 41, 42] suggesting that they have been present in Europe for millions of years.

To explore divergences of lineages at a wide timespan, we analyzed one mitochondrial and two nuclear markers with different substitution rates. The mitochondrial COI gene is widely used for barcoding but previous studies demonstrated high intraspecific variance in Collembola [43, 44]. In order to resolve divergences deeper in time we added two nuclear markers that evolve at a lower rate than COI. The D3-D5 loop of 28S rDNA is a useful species marker for soil-living animals [45] but also exhibits intra-individual variance in Collembola [46, 47]. Further, we included the nuclear protein-coding gene Histone 3 with an expected mutation rate intermediate between COI and the 28S rDNA fragment (D3-D5 loop). For estimating divergence times of genetic lineages in absence of fossil records of the investigated species, we applied the general mutation rate for arthropods of COI, ranging from 1.5–2.3 % sequence divergence per million years [48, 49], the mutation rates of the two nuclear genes are unknown and were estimated by BEAST [50] in a combined alignment.

Assuming that soil buffered Quaternary temperature extremes, Collembola presumably survived in local patches in central Europe and expanded from these patches. Accordingly, endemic haplotypes are present in central and northern Europe. Further, we expected different genetic lineages to coexist locally due to expansions from isolated central European refuge populations. Due to more extensive survival during Quaternary glaciations of Collembola in southern Europe, the local molecular variance and genetic distances within populations from southern Europe should be higher than those within central and northern European populations.

## Methods

### Sampling of animals and DNA extraction

Leaf litter including humus layers from about two square meters of deciduous and coniferous forests were collected in 19 locations in 13 countries in Europe, including northwest and middle-west Russia, and the Pleistocene refuge areas of the Balkan (Montenegro, Serbia, Croatia, Greece), Italy and Spain, covering a geographic sampling range of 4,500 km on the east–west and ~4,000 km on the north–south axis, extending other studies on European Collembola by including countries from the Mediterranean to Scandinavia and western Eurasia. Animals were extracted by heat [51], collected in 96 % EtOH and stored at−20 °C until further analyses. For species identification specimens were sorted under a dissecting microscope and determined by light microscopy following Hopkin [52]. If possible, five individuals per species and sampling location were sequenced (Table 1). In preliminary analyses we sequenced 12–20 individuals per species from 8 sampling locations and genetic variance within locations was very low, with uniformly a single genetic lineage dominating sampling locations. After these preliminary analyses we decided to restrict the sample size per sampling location to five individuals because for this study the dominant haplotypes are important to analyse genetic structure. Analysing more individuals per sampling location would include rare haplotypes and result in uneven sample sizes due to differences in natural abundance, which varied strongly from very few to hundreds of individuals per sampling location for all species.

**Table 1:** Summary of sampling sites of three species of Collembola sampled form forests across Europe

	Country	Location	Abbreviation	*Ceratophysella*	*Folsomia*	*Isotomiella*	coordinates
				*denticulata*	*quadrioculata*	*minor*	(N, E)
North	Estonia	Tallinn	EE	–	5	–	59.44° 24.69°
	Norway	Rod	NO	5	5	5	59.07° 10.23°
	Russia	Letnerechenskiy	RU1	–	4	5	64.27° 34.44°
		Nischni Novgorod	RU2	5	–	–	56.37° 43.98°
Central	Austria	Tirol, Sonnenberg Alm	AT1	–	–	5	47.46° 12.24°
		Hittisau	AT2	5	–	–	47.46° 9.95°
		Holzgau	AT3	–	3	–	47.29° 10.33°
	France	Chartreuse	FR	5	5	4	45.42° 5.81°
	Germany	Solling, Neuhaus	DE	5	5	4	51.71° 9.64°
	Poland	Warsaw	PL	4	–	4	52.33° 20.76°
South	Croatia	Sljeme	HR	3	4	4	45.90° 15.95°
	Greece	Chrysovitsi	GR	5	5	5	37.56° 22.20°
	Italy	Felitto	IT1	–	5	5	40.37° 15.22°
		Berceto	IT2	3	–	–	44.50° 10.00°
	Montenegro	Bar	ME	5	5	5	42.13° 19.09°
	Serbia	Markovac	RS	5	5	–	44.22° 21.09°
	Spain	Oviedo	ES1	–	–	5	43.36° -6.00°
		Ponga	ES2	4	–	4	43.19° -5.16°
		Martiartu	ES3	–	5	–	43.20° -2.90°
		Total no. of individuals.		54	56	55	

Abbreviations of sampling locations and number of individuals sequenced for this study are listed. Sampling locations are grouped into north, central and south European regions

Genomic DNA was extracted from single individuals of *C. denticulata* (*n* = 54)*, F. quadrioculata* (*n* = 56) and *I. minor* (*n* = 55) using the DNeasy® Blood and Tissue Kit (Qiagen, Hilden, Germany) following the manufacturer’s protocol for animal tissue. Purified DNA was eluted in 30 μl buffer AE and stored at−20 °C until further preparation. PCRs of the nuclear Histone 3 and 28S rDNA (D3-D5 region) and the mitochondrial COI gene were performed in 25 μl volumes containing 12.5 μl SuperHot Taq Mastermix (Genaxxon Bioscience GmbH, Ulm, Germany) with 1.5 μl of each primer (10 pM), 4.5 μl H_2_O, 2 μl MgCl_2_ (25 mM) and 3 μl template DNA. Primers and PCR programs are given in Additional file 2: Table S1. Positive PCR products were purified with the QIAquick PCR Purification Kit (Qiagen, Hilden, Germany) following the manufacturer’s protocol and sent for sequencing to the Göttingen Genome Laboratory (Institute for Microbiology and Genetics, Georg August University of Göttingen). All sequences are available at NCBI GenBank (KF684371-KF684865, Additional file 2: Table S2). DNA was extracted from entire specimens but secondary vouchers (same morphological species from the same population) were deposited at our collections at J.F. Blumenbach Institute of Zoology and Anthropology, Georg August University Göttingen, Germany. For molecular clock analyses, COI and 28S rDNA sequences of outgroup taxa from the same family, congeneric species, and the same species from additional, mainly non-European, locations were downloaded from NCBI and BOLD databanks (Additional file 2: Table S3).

### Phylogenetic analyses, divergence time estimation and population structure

Sequences were edited and corrected with Sequencher 4.10 (Gene Codes Corporation, USA), coding nucleotide sequences (H3 and COI) were translated into protein sequences using the standard and invertebrate mitochondrial codes implemented in Sequencher. For each species, nucleotide and protein sequences, were aligned separately and combined (as concatenated nucleotide matrix of three genes) with Clustal W [53] implemented in BioEdit 7.0.1 [54].

The best fit model of sequence evolution for each alignment (COI, 28S, H3, combined matrix) was inferred with to the hLRT in TOPALi v2.5 [55] using the PHYML algorithm. Phylogenetic trees were calculated with RAxML v7.0.3 [56] and MrBayes v3.1.2 [57]. For Maximum likelihood analyses the model of sequence evolution was GTR + I + G (all four alignments) and 10,000 bootstrap replicates were calculated. For Bayesian Inference lset parameters were nst = 6, rates = invgamma (all four alignments), the MCMC (Markov Chain Monte Carlo) chains were run for ten million generations that were sampled every 1,000th generation. For the 10,000 sampled generations a burnin of 2,500 was used, eliminating the first 25 % of the remaining generations. In the absence of fossil or biogeographic calibration points for the investigated species in Europe, a strict molecular clock for the COI nucleotide alignment was applied in BEAST v1.7.4 [58]. For constructing trees we used the Yule Process [59] as preliminary analyses indicated quicker convergence and higher probabilities and likelihoods than coalescent tree priors. However, topologies with different tree priors did not vary. The Yule Process also is more appropriate for the genetically highly diverged lineages as the substitution rate among branches is more variable than with coalescent priors. We used the widely adopted mutation rate for COI in arthropods of 2.3 % pairwise sequence divergence per million years, corresponding to a rate of 0.0115 [48, 49].

Convergence of the MCMC chain after 600 million generations (sampled every 60,000th generation) with a burnin of 25 % was confirmed using Tracer v1.4 [60]. Divergence estimates were calculated with three datasets: (1) all COI and 28S sequences of this study and NCBI combined, with strict clock settings for COI (fixed rate of 0.0115) and estimated rates for 28S, (2) all COI sequences obtained in this study with a strict clock (fixed rate of 0.0115), and (3) all COI sequences of this study extended with sequences from non-European countries obtained from NCBI and BOLD databanks with a strict clock (fixed rate of 0.0115). The extended datasets (2) and (3) included additional sequences from Antarctica, Australia, Canada, Chile, New Zealand, South Africa and northern France and a more detailed outgroup sampling for better estimation of the substitution rate. Outgroups covered additional species within the respective genera and families, i.e. *Hypogastrura* (five species), *Xenylla grisea* and *Gomphiocephalus hodgsoni* for Hypogastruridae and *Folsomia* (three species), *Parisotoma notabilis, Anurophorus septentrionalis* and *Isotoma* (three species) for Isotomidae (Additional file 2: Table S3). For *F. quadrioculata* and *I. minor*, non-European 28S sequences of the D3-D5 region were not available and the combined datasets were extended only with the above mentioned outgroup taxa. The outgroup settings of the two isotomid species were identical, except for five additional sequences of the parthenogenetic species *Parisotoma notabilis*, to account for variance in the substitution rate due to the reproductive mode of *I. minor*.

Median-joining haplotype networks for the nucleotide datasets of COI, H3, 28S and the concatenated dataset were generated for all three species using the program Network 4.6 (Fluxus Technology, Suffolk, Great Britain). Molecular variance (AMOVA) within and between populations and isolation by distance (Mantel test) of all three genes (uncorrected p-distances) were analyzed separately in ARLEQUIN [61] with 20,000 permutations. To infer the molecular divergence times of *C. denticulata*, *F. quadrioculata* and *I. minor*, we generated a phylogenetic tree of 10 families with 23 genera and eight outgroup taxa including the taxa studied by D’Haese [62], using 28 COI sequences available at NCBI and a strict molecular clock in BEAST as described above.

## Results

### Population structure

Intraspecific variance of the three genes was similar in the three species. For *C. denticulata* the nucleotide alignments of COI, H3 and 28S had 240, 75 and 45 variable positions, respectively. Respective values for *F. quadrioculata* were 234, 65 and 2 and for *I. minor* 253, 50 and 15.

Isolation by distance was rejected as not being significant for all datasets and all species; however, genetic variance among populations was high, explaining 96–97 % (*C. denticulata*), 87–88 % (*F. quadrioculata*) and 92–99 % (*I. minor*) of the genetic variance of COI, H3 and the concatenated dataset (COI and H3; Table 2). Genetic distances between populations were very high (Table 3), ranging for COI between 14 and 20 % in *C. denticulata* and *I. minor* and 11–17 % in *F. quadrioculata*. Within population distances were zero or very low (<2 %). The only exceptions were one population of *C. denticulata* (Croatia: H3, 5.5 %), two populations of *F. quadrioculata* (Croatia: COI, 8 % and Greece: COI, 9.5 %; H3, 4.3 %) and two populations of *I. minor* (Russia: COI, 5.8 % and Germany: COI, 4.9 %) in which distinct COI and H3 lineages co-occurred. Genetic distances between and within regions (COI and H3) were highest in southern Europe and decreased towards the north, but were lowest within central Europe (Table 4). Between several populations in central and northern Europe genetic distances were lower than average (Table 5). Distances were low for H3 but high for COI between populations from Norway, Germany and France (*F. quadrioculata;* 14 %), from Montenegro and Italy (*C. denticulata*, 16 %), and from Norway and Spain (*I. minor*; 14 %).

**Table 2:** Variance partitioning among and within sampling locations (AMOVA) of three springtail species (*Ceratophysella denticulata*, *Folsomia quadrioculata* and *Isotomiella minor*) sampled across Europe based on sequence variance of the mitochondrial COI gene, the nuclear H3 gene and a concatenated dataset of three genes (COI, H3 and 28S)

		COI		H3		conc (COI + H3 + 28S)	
	Source of variation	Among pop.	Within pop.	Among pop.	Within pop.	Among pop.	Within pop.
*C. denticulata*	d.f.	11	42	11	42	11	42
	sum of squares	2,831.46	77.67	674.86	25.07	3,980.26	103.48
	variance components	56.94 Va***	1.85 Vb***	13.54 Va***	0.60 Vb***	80.07 Va***	2.46 Vb***
	% variation	96.85	3.15	95.78	4.22	97.01	2.99
	fixiation indices	Fst 0.969***		Fst 0.958***		Fst 0.970***	
*F. quadrioculata*	d.f.	11	44	11	44	11	44
	sum of squares	2,413.07	311.2	486.74	53.6	2,867.11	364.8
	variance components	45.57 Va***	7.07 Vb***	9.24 Va***	1.22 Vb***	54.16 Va***	8.29 Vb***
	% variation	86.57	13.43	88.35	11.65	86.72	13.28
	fixiation indices	Fst 0.866***		Fst 0.883***		Fst 0.867***	
*I. minor*	d.f.	11	43	11	43	11	43
	sum of squares	2,784.45	193.35	453.47	4.95	3,346.90	198.3
	variance components	54.30 Va***	4.50 Vb***	8.98 Va***	0.12 Vb***	65.45 Va***	4.61 Vb***
	% variation	92.35	7.65	98.73	1.27	93.42	6.58
	fixiation indices	Fst 0.923***		Fst 0.987***		Fst 0.934***	

Asterisks indicate significant differences at *p* < 0.05, d.f., degrees of freedom

significant levels: * *p*<0.05, ** *p*<0.01, *** *p*<0.001

**Table 3:** Summary of genetic distances of COI and H3 in three European Collembola species (*Ceratophysella denticulata*, *Folsomia quadrioculata* and *Isotomiella minor*)

(*a*)														
Between populations									Within populations					
			*C. denticulata*		*F. quadrioculata*		*I. minor*		*C. denticulata*		*F. quadrioculata*		*I. minor*	
	Location		COI	H3	COI	H3	COI	H3	COI	H3	COI	H3	COI	H3
North	EE	Estonia			14 ± 1	5 ± 2					0.0	1.39		
	NO	Norway	16 ± 4	7 ± 2	15 ± 2	5 ± 2	15 ± 1	4 ± 2	0.06	0.0	0.06	0.0	0.11	0.0
	RU1	Russia	17 ± 3	7 ± 2	14 ± 1	5 ± 2	14 ± 6	4 ± 3	0.06	0.0	0.99	0.0	5.84	0.16
Central	AT	Austria	15 ± 5	7 ± 3	13 ± 3	5 ± 2	15 ± 7	4 ± 3	0.0	0.0	0.0	0.0	0.0	0.0
	FR	France	15 ± 5	7 ± 3	14 ± 2	4 ± 2	17 ± 1	5 ± 2	0.73	0.0	1.02	0.0	0.21	0.0
	DE	Germany	15 ± 5	6 ± 3	13 ± 3	5 ± 2	15 ± 5	5 ± 3	1.16	0.0	0.06	0.11	4.87	0.13
	PL	Poland	15 ± 5	7 ± 3			15 ± 7	5 ± 3	1.03	0.0			0.0	0.0
South	HR	Croatia	14 ± 4	7 ± 2	15 ± 1	6 ± 1	18 ± 1	5 ± 2	0.09	5.53	8.04	0.0	1.6	0.53
	GR	Greece	19 ± 1	7 ± 1	15 ± 1	7 ± 1	17 ± 5	4 ± 2	1.27	0.48	9.51	4.33	0.0	0.0
	IT	Italy	17 ± 2	10 ± 1	14 ± 1	4 ± 2	17 ± 5	4 ± 2	0.0	0.0	0.0	0.0	0.06	0.0
	ME	Montenegro	17 ± 2	9 ± 2	15 ± 1	7 ± 1	18 ± 1	5 ± 2	0.39	0.11	1.35	0.0	0.71	0.0
	RS	Serbia	17 ± 3	8 ± 2	15 ± 1	6 ± 1			0.0	0.0	1.97	1.23	0.0	0.0
	ES1	Spain					18 ± 1	9 ± 0					1.86	0.0
	ES2	Spain	19 ± 1	10 ± 1			17 ± 1	5 ± 1	1.32	0.0			0.07	0.0
	ES3	Spain			16 ± 1	8 ± 2					1.21	0.11		

Mean *p*-distances are in percent with standard deviation (*a*) between and within populations, (*b*) geographic regions, and of (*c*) geographic clusters with exceptionally low genetic distances

**Table 4:** Summary of genetic distances of COI and H3 in three European Collembola species (*Ceratophysella denticulata*, *Folsomia quadrioculata* and *Isotomiella minor*). Mean *p*-distances are in percent with standard deviation (*a*) between and within populations, (*b*) geographic regions, and of (*c*) geographic clusters with exceptionally low genetic distances

(*b*)																		
COI											H3							
	*C. denticulata*			*F. quadrioculata*			*I. minor*			*C. denticulata*			*F. quadrioculata*			*I. minor*		
Region	N	C	S	N	C	S	N	C	S	N	C	S	N	C	S	N	C	S
North (N)	10			9			11			4.2			2.7			2.8		
Central (C)	14	9		13	7		13	9		5.7	2.9		3.1	1.6		3.2	2.5	
South (S)	17	17	16	16	16	14	18	18	14	8.0	8.7	7.4	6.2	6.0	5.8	5.2	5.5	4.5

**Table 5:** Summary of genetic distances of COI and H3 in three European Collembola species (*Ceratophysella denticulata*, *Folsomia quadrioculata* and *Isotomiella minor*). Mean *p*-distances are in percent with standard deviation (*a*) between and within populations, (*b*) geographic regions, and of (*c*) geographic clusters with exceptionally low genetic distances

(*c*)						
	*C. denticulata*		*F. quadrioculata*		*I. minor*	
Geographic cluster	COI	H3	COI	H3	COI	H3
NO-ES2					14.6	0.5
ME-IT	16	5.7				
GR-IT					1.9	0.3
AT-FR	0.3	2.7				
AT-DE			5.2	2	7.3	0.5
RU2-RS	9.8	2.9				
AT-PL-RU1					0.9-4.3	0.6-1
NO-DE-FR			14.1-14.7	1.7-2.7		
HR-DE-PL-NO	6.3-10.5	2.4-7.9				

The nuclear 28S rDNA (D3-D5 region) had the lowest genetic variance with little (6.2 % and 1.8 % in *C. denticulata* and *I. minor*, respectively) or almost no variation between populations (0.2 % for *F. quadrioculata*). Accordingly, the network analysis separated only haplotypes of *C. denticulata* into distinct geographic clusters (Additional file 1: Figure S1).

### Phylogenetic analyses

**Fig. 1:**
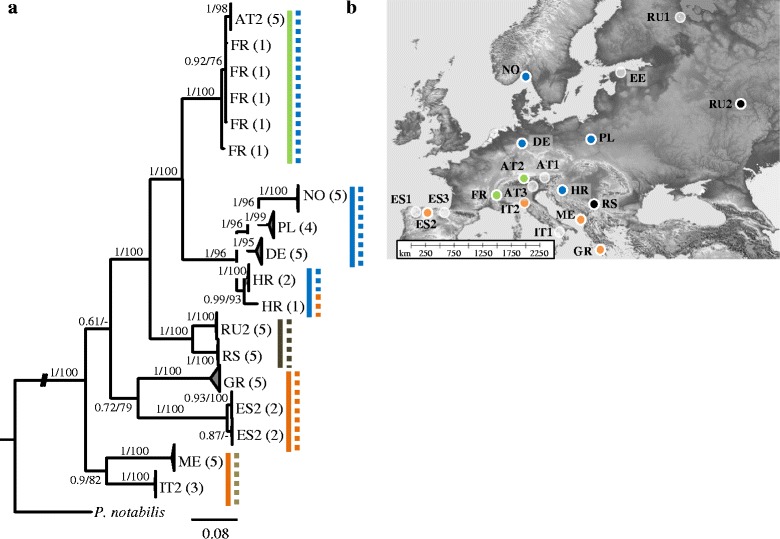
Relationships of lineages of *Ceratophysella denticulata* in Europe derived from (**a**) Bayesian analysis of sequence data of three genes; distinct genetic lineages are indicated by solid (COI) and dashed (H3) lines. **b** Sampling locations and geographic distribution of COI lineages; white sampling locations are not represented by this species. Terminal clades in the phylogenetic tree have been collapsed and numbers of individuals included in each clade are indicated by numbers in brackets. Numbers on nodes are posterior probabilities (Bayesian Inference) and bootstrap values (Maximum Likelihood)

**Fig. 2:**
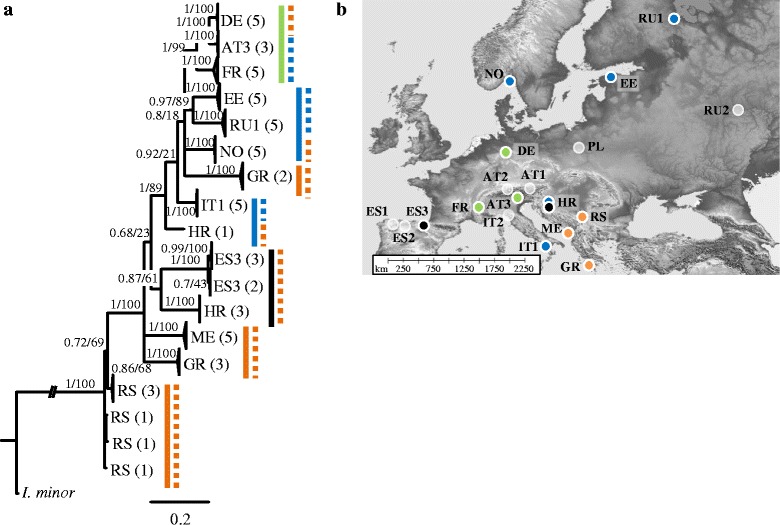
Relationships of lineages of *Folsomia quadrioculata* in Europe derived from (**a**) Bayesian analysis of sequence data of three genes; distinct genetic lineages are indicated by solid (COI) and dashed (H3) lines. **b** Sampling locations and geographic distribution of COI lineages; white sampling locations are not represented by this species

**Fig. 3:**
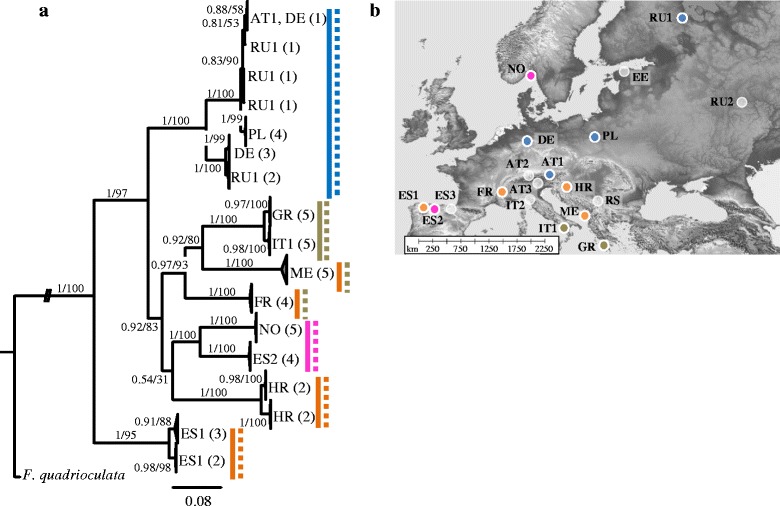
Relationships of lineages of *Isotomiella minor* in Europe derived from (*a*) Bayesian analysis of sequence data of three genes; distinct genetic lineages are indicated by solid (COI) and dashed (H3) lines. (*b*) Sampling locations and geographic distribution of COI lineages; white sampling locations are not represented by this species

Phylogenetic trees based on the combined matrix gave the best resolution and statistical support for internal and terminal nodes (Figs. 1, 2 and 3). Topologies of ML and BI trees were similar with nearly all sampling locations clustering in separate clades with high support (pp: 0.97-1; bp: 95–100). Populations from southern European countries comprised a number of isolated COI and H3 lineages which derived early in each of the trees. Central and northern European populations were more homogeneous with shorter and more derived branches. Common patterns in each of the three species were the presence of one or two closely related phylogeographic clades with a wide distribution range across central and north-east Europe (blue and green lineages). Sampling locations in the Mediterranean were not covered by phylogeographic clades but rather by genetically distinct and phylogenetically isolated lineages (orange lineages). Interestingly, both isotomid species had phylogenetically related lineages with disjunct distributions. In *F. quadrioculata*, individuals from Spain and Croatia (black lineage) were monophyletic in the single gene phylogenetic trees and carried identical 28S sequences. In *I. minor*, individuals from Norway and Spain had distinct mitochondrial haplotypes, but nearly identical H3 and 28S alleles. Coexistence of genetic lineages did not occur in each of the three species except for one population from Croatia of *F. quadrioculata*. Different to the other species, *C. denticulata* had a central-eastern phylogeographic clade (black lineage) but the geographic range and spatial coherence of this lineage remains open due to the limited number of sampling locations.

Haplotype diversity of the mitochondrial gene was generally high in the three investigated species (Table 6) but most haplotypes differed in only a few positions. Haplotype networks of the COI and H3 gene were complex in each of the species as the majority of populations were separated by >10 mutation steps (Additional file 1: Figures S1-S3). For simplifying the dataset, we grouped the COI haplotypes and H3 and 28S alleles into lineages according to clades with very high [posterior probabilities (pp): 1, bootstrap (bs): 100] or high node support (pp: 0.97-99, bs: 89–100) in the phylogenetic trees based on single gene alignments (Additional file 1: Figures S4–S5).

### Estimation of divergence times

**Fig. 4:**
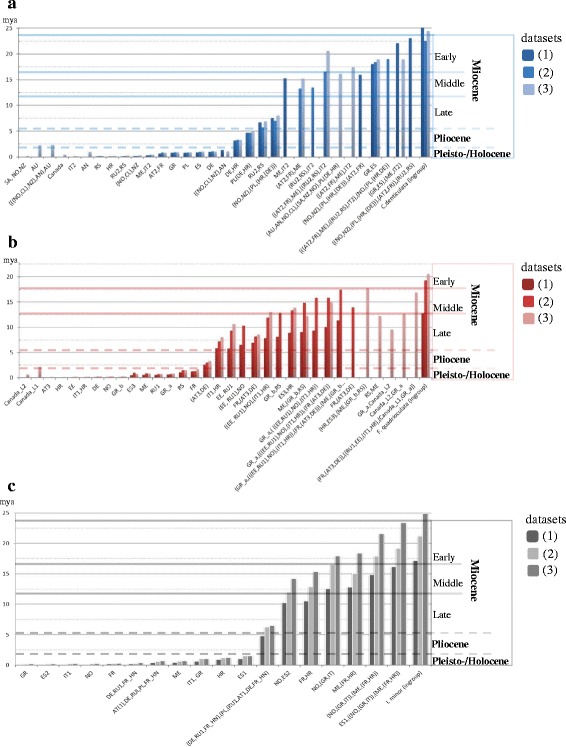
Comparison of molecular divergence time estimates of three datasets of (**a**) *Ceratophysella denticulata*, (**b**) *Folsomia quadrioculata* and (**c**) *Isotomiella minor* calculated with BEAST. Adjacent columns indicate divergence times per node, the left column (dark color) represents dataset 1 (28S + COI), the central column (light color) refers to dataset 2 (COI only this study) and the right column (intermediate color) dataset 3 (COI with additional non-European taxa). Sampling locations included in nodes are indicated on the x-axis, for topology of the phylogenetic trees see (Additional file 1: Figures S7 and S9-11)

Trees for molecular divergence estimates calculated with COI in BEAST differed only slightly from those generated with the combined matrix (Additional file 1: Figure S6). All populations and clades that comprised larger geographic areas were recovered, only positions of populations from Italy (*C. denticulata*), Greece (*F. quadrioculata*), Croatia (*I.minor*) and Montenegro (*C. denticulata* and *I. minor*) differed from the trees calculated with the combined matrices. Divergence estimates of major lineages fell into the Miocene (23.03-5.3 mya) in each of the three species, considerably predating Quaternary glaciations (1.8 my to present). Results were consistent among the three datasets, demonstrating robustness of the molecular clock analyses (Fig. 4).

Three major radiation events can be extrapolated from the datasets of the three species (Fig. 4, Additional file 1: Figure S6). First, large phylogeographic clades of lineages now present in central and north-east Europe separated from south European lineages during the Early and Middle Miocene (23.03-11.6 mya); second, clades in central and northern Europe separated during the Late Miocene (11.6-5.4 mya), and third, populations, i.e. sampling locations, radiated during the Pleistocene (0.011-1.8 mya). Divergence estimates of the COI datasets were consistent, divergences in dataset (2) without additional outgroups were even more conserved, i. e. lineages in general were younger. In the two isotomid species (*F. quadrioculata* and *I. minor*) divergence estimates of larger phylogeographic clades were younger in dataset (1) (combined COI and 28S) compared to datasets (2)–(3) (COI only), with predominantly Middle Miocene origin (16–11.5 mya). However, this probably is an artefact of the 28S gene which is strongly conserved among genera of *Folsomia* (H. von Saltzwedel, unpublished data) and probably other Isotomidae. In general, divergences of clades and populations were more recent in *F. quadrioculata* than in the other two species but still concentrated in the Miocene, independent of the dataset analyzed.

The phylogeographic dataset can only estimate the molecular age of European lineages of the respective species, which ranged between 22.5 and 25.8 my for *C. denticulata*, between 12.8 and 20.6 my for *F. quadrioculata* and between 17.2 and 24.9 my for *I. minor*, depending on the dataset analyzed. Estimated divergence times of the three Collembola species based on a phylogeny of 28 COI sequences can infer the ages of the respective genera, which were about twice as old with 48 ± 13 my for *C. denticulata*, 56 ± 14 my for *F. quadrioculata* and 49 ± 15 my for *I. minor* (Additional file 1: Figure S7). The age of the species must be between these two age estimates. As both analyses do not conflict with the fossil record, we take this as support for our molecular clock analyses and the Miocene origin of large phylogeographic clades in European Collembola.

## Discussion

This study investigated the genetic structure of Collembola at a pan-European scale. The results suggest three largely consistent phylogeographic patterns of the three investigated species. First, populations of Collembola in Europe are highly structured with high genetic variance between, but low variance within populations. Second, molecular divergence estimates and genetic distances suggest that common ancestors of present day populations in central and southern Europe probably persisted in isolated populations for millions of years. This is in agreement with our hypothesis that Quaternary climate changes were less severe for soil animals than for aboveground animals. Third, Miocene divergences dominate among geographic lineages indicating that diversification events correlate with wet and warm climate, and a biome change in central and eastern Europe from forest to grassland. The presence of the three species of Collembola in Europe during the Miocene is supported by their estimated Eocene origin that also correlates with the fossil record from Baltic amber [3–6].

In each of the three species, the majority of sampling locations were dominated by a single haplotype, suggesting that only few early colonizing individuals founded the populations which grew and expanded rapidly thereby preventing invasion of other lineages. This is conform to the founder-takes-it-all process [63] resulting in low genetic variance within but high variance between populations. Molecular divergence estimates of populations substantially predated Quaternary glaciations and originated in the Miocene. Long-term persistence of distinct genetic lineages without mixing of populations presumably is related to low mobility and high local abundances of springtails.

Strong molecular differentiation between populations indicates the existence of cryptic species. Indeed, recent molecular studies suggest that due to the widespread occurrence of cryptic species the number of Collembola species may be magnitudes higher than current estimates based on morphology [64, 65]. However, without evidence of differences in physiological, feeding, or mating preferences, we refrain from discussing potential cryptic species but rather consider them as species with high intraspecific genetic variance in COI. High genetic variation in COI have been reported from other arthropods in particular those living in soil, including Collembola [28, 43, 44, 65], oribatid mites [24, 66, 67] and Opilionida [68]. In the sexual species *C. denticulata* genetic variance in nuclear genes was high but genetic distances within the slowly evolving 28S gene still were below the intra-lineage threshold previously estimated for this species [46].

Genetic distances between and within regions were high in the south compared to distances between and within the central and northern European regions. This suggests that Collembola follow the “southern richness and northern purity” scenario of recolonization of central and northern Europe [69]. Lower distances within the central region suggest more recent expansion of populations or recent colonization of genetic lineages from east Europe or central Asia. However, molecular divergence time estimates of the three Collembola species indicate pronounced radiations into geographic lineages during the Miocene (20–5 mya). These divergences are considerably older than for most aboveground animals, suggesting that extinctions of populations during the Quaternary and recolonization of central Europe thereafter [70] had only minor effects on the population structure of present day Collembola populations.

The Miocene was warm and humid resembling subtropical climate with central Europe separating later into marine and continental climatic zones along west–east gradients with seasonality increasing to the east [71]. For little sclerotized arthropods, susceptible to desiccation such as Collembola, the warm climate and high precipitation during the Miocene potentially facilitated large scale expansion. Accordingly, our divergence estimates indicate that southern and central European lineages separated during the Mid Miocene Climate Optimum (MMCO, 17–14 mya) when precipitation and temperature were higher than in the Early Miocene [72]. Increasing seasonality and decreasing precipitation during the Miocene also changed the vegetation across Europe favoring the spread of deciduous forests, open woodlands and grassland vegetation in eastern and southern Europe, which expanded considerably during the Late Miocene (Messinian, 7.2-5.3 mya) [71, 73]. Interestingly, clades of *C. denticulata* and *F. quadrioculata* that cover the geographic regions from south-east, central-east and central Europe (Croatia, Russia, Serbia Austria, Germany, Poland) were dated to be of Late Miocene origin. The lower than average genetic distance in these clades suggests that a single or a few lineages expanded into these areas, possibly due to fragmentation of forest habitats into open woodlands, causing local extinctions or adaptations. In contrast, lineages of the parthenogenetic species *I. minor* were less separated into geographic regions, but rather split continuously from a ubiquitous and widespread ancestral lineage. This is also reflected by the equal distances of haplotypes in the network of the nuclear marker (28S), indicating that no region is more ancestral to others and suggesting separation of these lineages from a common ancestor. Accordingly, the two closely related (28S and H3) but geographically separated populations of Norway and eastern Spain likely represent relict populations that separated from an ancestral lineage about 12 mya and survived in isolated patches. Related lineages either went extinct or were not sampled. Further, divergences into geographic lineages mainly occurred during the Early and Middle Miocene and Pleistocene in *I. minor*, with only one divergence within the central European clade during the Late Miocene. In contrast, divergences of central European lineages of *C. denticulata* and *F. quadrioculata* predominantly took place several million years later in the Late Miocene and Early Pliocene.

Collembola are fast colonizers of newly formed and young habitats such as glacier forelands [74], but the geographic origin of these founder populations are unknown. The distinctness of populations, including the most closely related populations from the east of Europe suggests a regional recruitment of colonizers from local source populations that survived in glacial refugia and consequently diverged from more distant populations for millions of years. A more detailed sampling likely would reveal even more divergent haplotypes providing a more coherent picture of distribution ranges of genetic lineages. Further, a more detailed sampling in north-east and central-east Europe may uncover if north European Collembola descended from geographically distant source populations in Eurasia or from genetically distinct source populations from within Europe.

Southern European populations were dominated by isolated lineages, supporting the existence of south European refuge areas but also suggesting that the Alps function as major dispersal barrier. Accordingly, regional extinctions in central Europe and rapid recolonization of empty habitats from the surrounding, rather than from southern refugia, likely resulted in the strong phylogeographic structure of populations north of the Alps. Strong genetic differentiation, little or no mixing between populations and ancient diversifications resemble the pattern reported for epigeic springtail species of the genus *Lepidocyrtus* in the Mediterranean basin [28] and the soil-living oribatid mite species *S. magnus* [24]. This indicates that evolutionary changes in soil are much slower and follow different trajectories as in aboveground invertebrates. However, coexistence of genetically distinct lineages in populations of the oribatid mite species *S. magnus* was more common than in the studied Collembola. This suggests that different factors regulate the establishment of populations among belowground invertebrates and that competition and early habitat colonization is more important for springtails than for oribatid mites [32, 74, 75].

## Conclusion

Similar to distribution patterns of European springtail species [76], we show that genetic structure within Collembola species also deviate from common aboveground patterns. Overall, the results suggest that populations of Collembola across Europe are highly structured and that diversifications into regional lineages predominantly occurred in the Miocene with these lineages still persisting*.* Divergence dates and geographic structure suggest that soil microarthropods are less affected by global and long-term climatic changes than aboveground animals and plants. Buffering of adverse climatic conditions, the presence of organic matter colonized by bacteria and fungi in soil, large population sizes and the ability to tolerate freezing conditions likely contributed to the lower responsiveness of soil microarthropods. This suggests that in soil evolutionary processes are slowed down compared to the aboveground system, rendering soil-living detritivore microarthropods “living fossils” with communities in central Europe reflecting Miocene rather than Quaternary diversification events.

## Additional files

Additional file 1: Figure S1. Map of all sampling points and approcimate sketches of extent of permanent ice shields (white filled areas) and borders of polar desert (red, dotted line) and permafrost (black, dashed line) climate during the Last Glacial Maximum (~20,000 years ago) that strongly shaped genetic and species richness above the ground in central and northern Europe (after Hewitt [69]). **Figure S2.** Haplotype Networks of *Ceratophysella denticulata*. **Figure S3.** Haplotype Networks of *Folsomia quadrioculata.* **Figure S4.** Haplotype Networks of *Isotomiella minor.* **Figure S5.** Bayesian phylogeny based on COI. **Figure S6.** Bayesian phylogeny based on H3. **Figure S7.** Molecular divergence estimates of dataset (2). **Figure S8.** Molecular phylogeny and divergence estimates of 28 species of Collembola based on COI calculated with BEAST. **Figure S9.** Molecular divergence estimates in *C. denticulata* of a) dataset (1) and b) dataset (3). *Outgroups: C. = Ceratophysella, H. = Hypogastrura, X = Xenylla, G = Gomphiocephalus*. **Figure S10.** Molecular divergence estimates in *F. quadrioculata* of a) dataset (1) and b) dataset (3). *Outgroups: F. = Folsomia, I. = Isotoma, C. = Cryptopygus, A. = Anurophorus.* **Figure S11.** Molecular divergence estimates in *I. minor* of a) dataset (1) and b) dataset (3). *Outgroups: F. = Folsomia, I. = Isotoma, C. = Cryptopygus, A. = Anurophorus*. (PDF 920 kb) Additional file 2: Table S1. a) Primer sequence of and b) PCR conditions for the ribosomal D3-D5 region of 28S rDNA, and the protein coding Histone 3 (H3) and COI genes. **Table S2.** DNA sequences available at GenBank. **Table S3.** Summary of additional locations and outgroup taxa included in the extended datasets (1) and (3) of the molecular clock analyses. Accession numbers from NCBI and BOLD databanks are listed. For some isotomiid species, the complete D3 fragment of 28S was not available, missing positions were filled with “?” in the alignments. (XLSX 17 kb)
