# Supplementary material for: Founder events and pre-glacial divergences shape the genetic structure of European Collembola species
Source: BMC Evol Biol. 2016 Jul 16;16:148. doi: 10.1186/s12862-016-0719-8 (PMC4947257; doi:10.1186/s12862-016-0719-8)
Supplement: Additional file 1: Figure S1. — Map of all sampling points and approcimate sketches of extent of permanent ice shields (white filled areas) and borders of polar desert (red, dotted line) and permafrost (black, dashed line) climate during the Last Glacial Maximum (~20,000 years ago) that strongly shaped genetic and species richness above the ground in central and northern Europe (after Hewitt [69]). Figure S2. Haplotype Networks of Ceratophysella denticulata. Figure S3. Haplotype Networks of Folsomia quadrioculata. Figure S4. Haplotype Networks of Isotomiella minor. Figure S5. Bayesian phylogeny based on COI. Figure S6. Bayesian phylogeny based on H3. Figure S7. Molecular divergence estimates of dataset (2). Figure S8. Molecular phylogeny and divergence estimates of 28 species of Collembola based on COI calculated with BEAST. Figure S9. Molecular divergence estimates in C. denticulata of a) dataset (1) and b) dataset (3). Outgroups: C. = Ceratophysella, H. = Hypogastrura, X = Xenylla, G = Gomphiocephalus. Figure S10. Molecular divergence estimates in F. quadrioculata of a) dataset (1) and b) dataset (3). Outgroups: F. = Folsomia, I. = Isotoma, C. = Cryptopygus, A. = Anurophorus. Figure S11. Molecular divergence estimates in I. minor of a) dataset (1) and b) dataset (3). Outgroups: F. = Folsomia, I. = Isotoma, C. = Cryptopygus, A. = Anurophorus. (PDF 920 kb) [file 12862_2016_719_MOESM1_ESM.pdf]

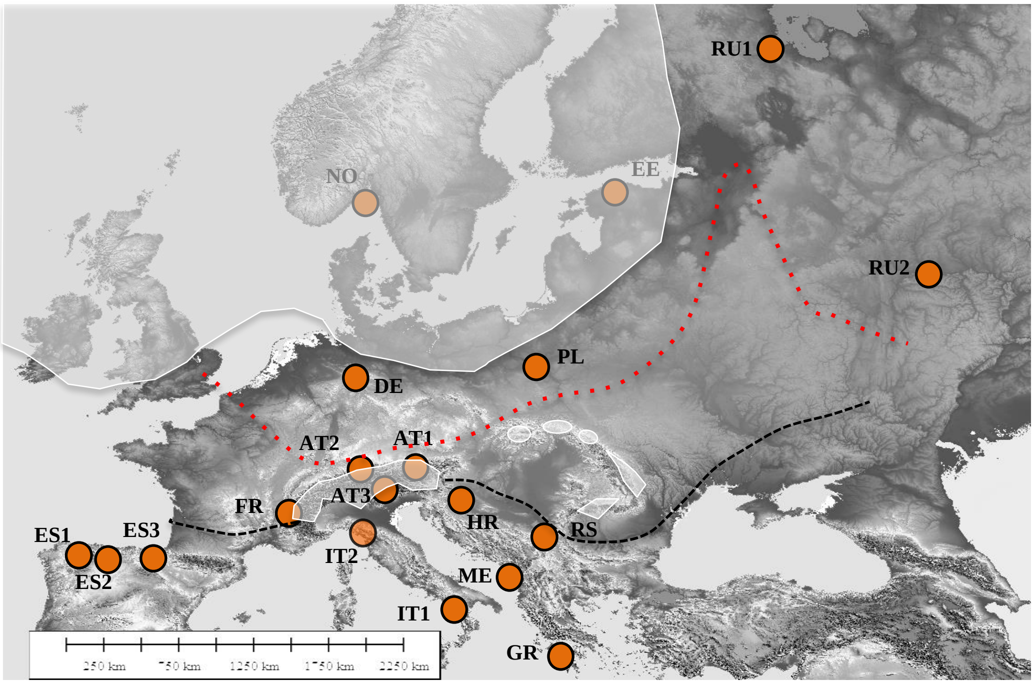

Fig. S1 Map of all sampling points and approcimate sketches of extent of permanent ice shields(white filled areas) and borders of polar desert (red, dotted line) and permafrost (black, dashed line) climate during the Last Glacial Maximum (~20,000 years ago) that strongly shaped genetic and species richness above the ground in central and northern Europe (after Hewitt 2001 [69]).

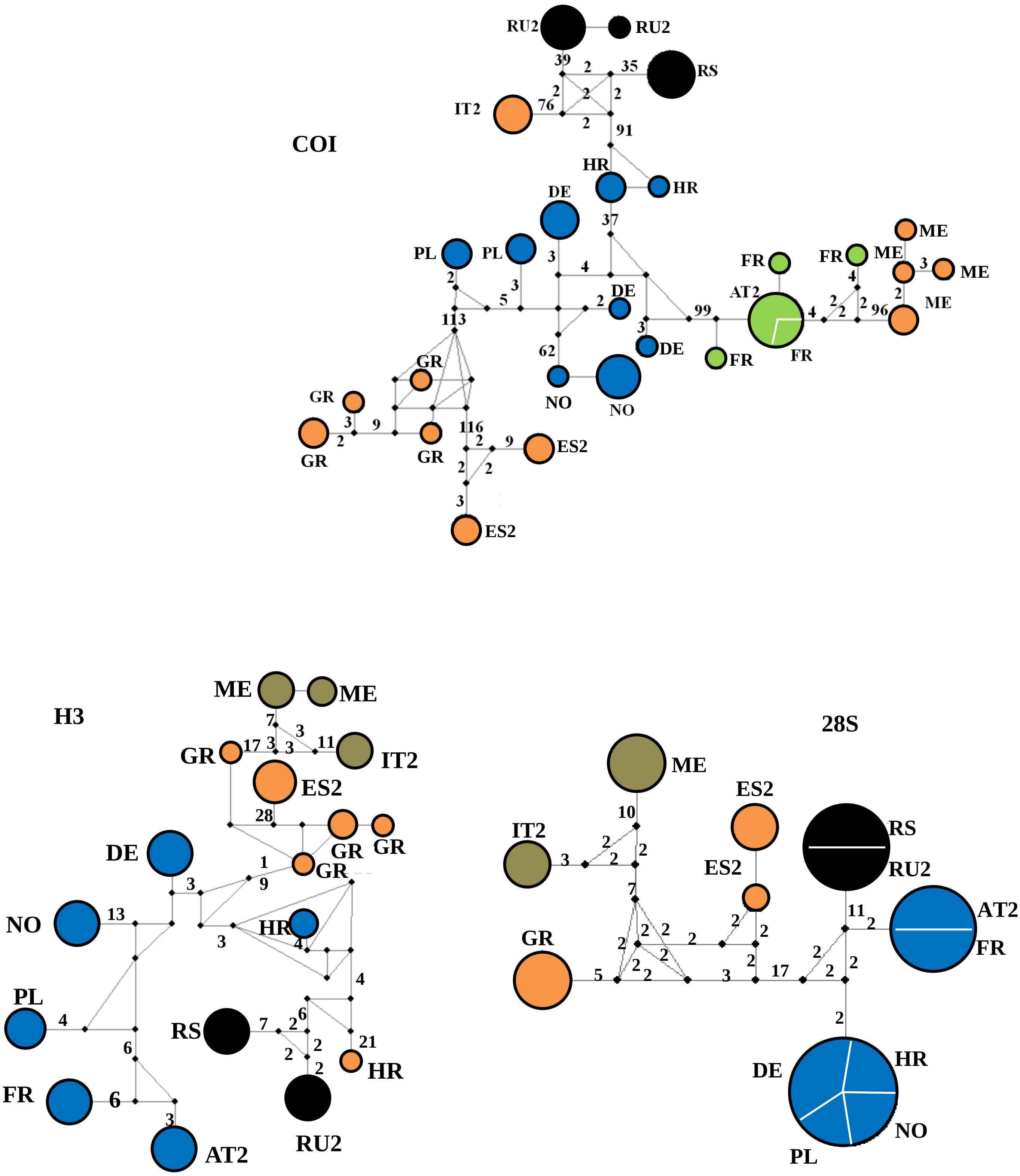

Fig. S2 Haplotype Networks of *Ceratophysella denticulata*

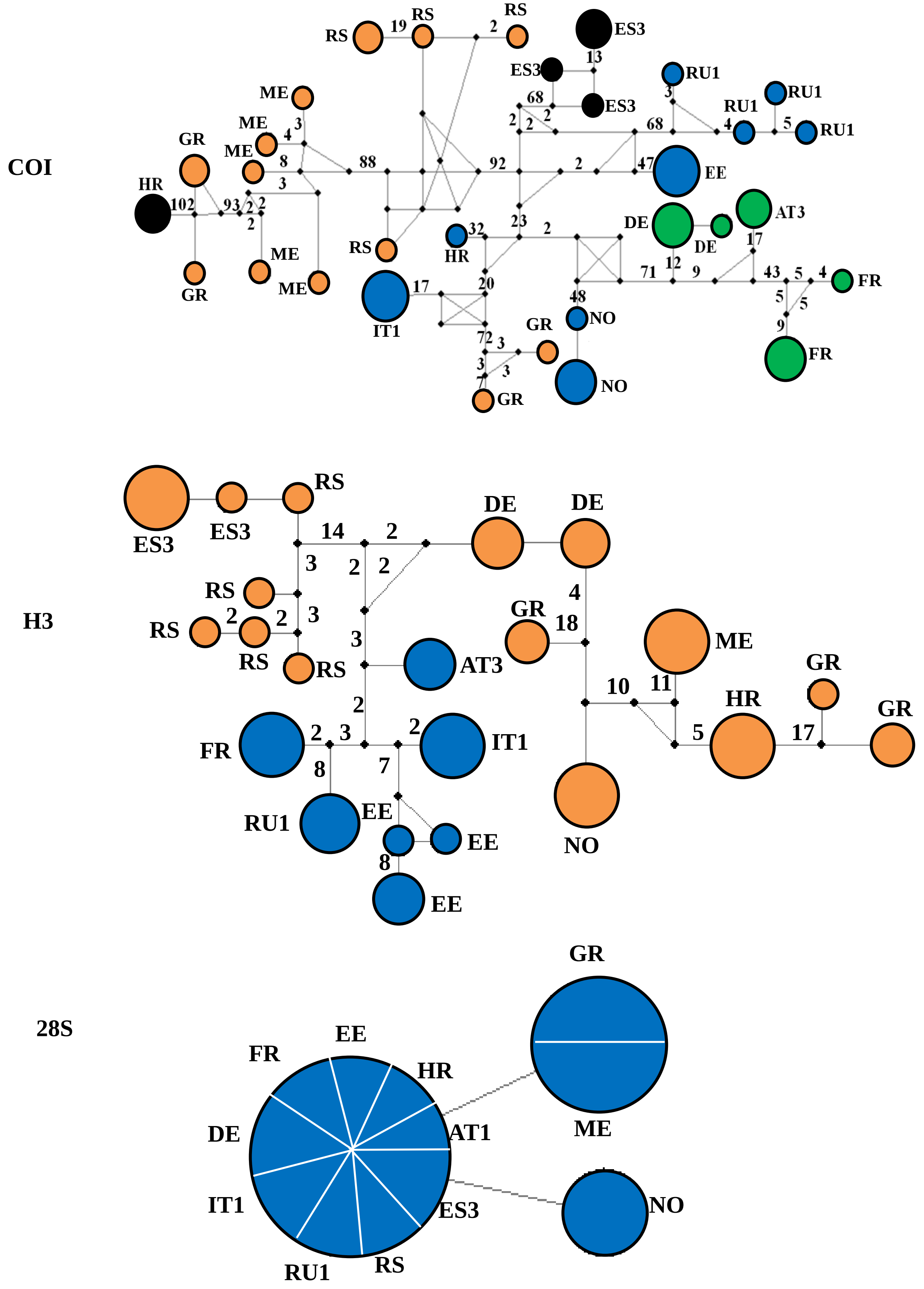

Fig. S3 Haplotype Networks of *Folsomia quadrioculata*

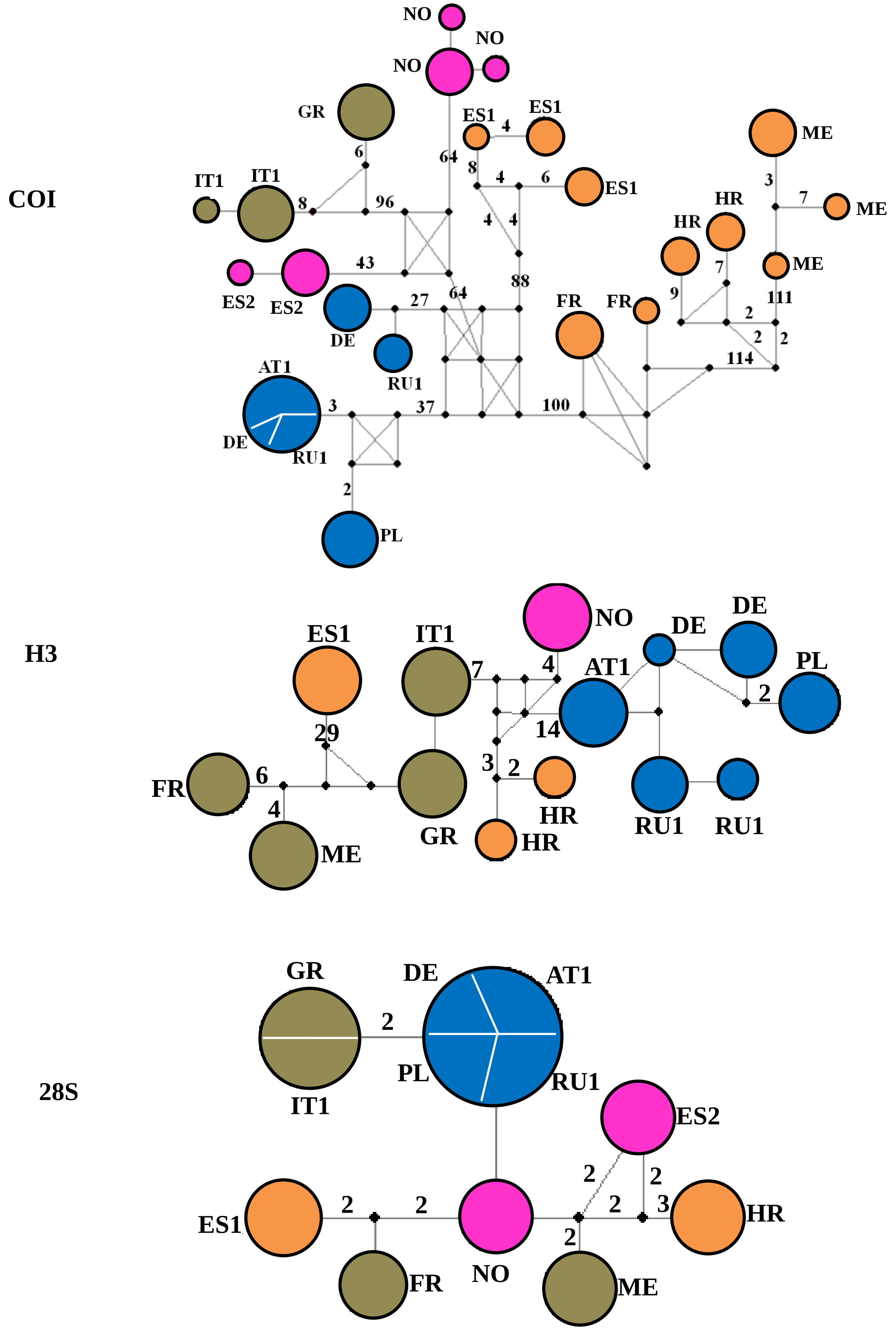

Fig. S4 Haplotype Networks of *Isotomiella minor*

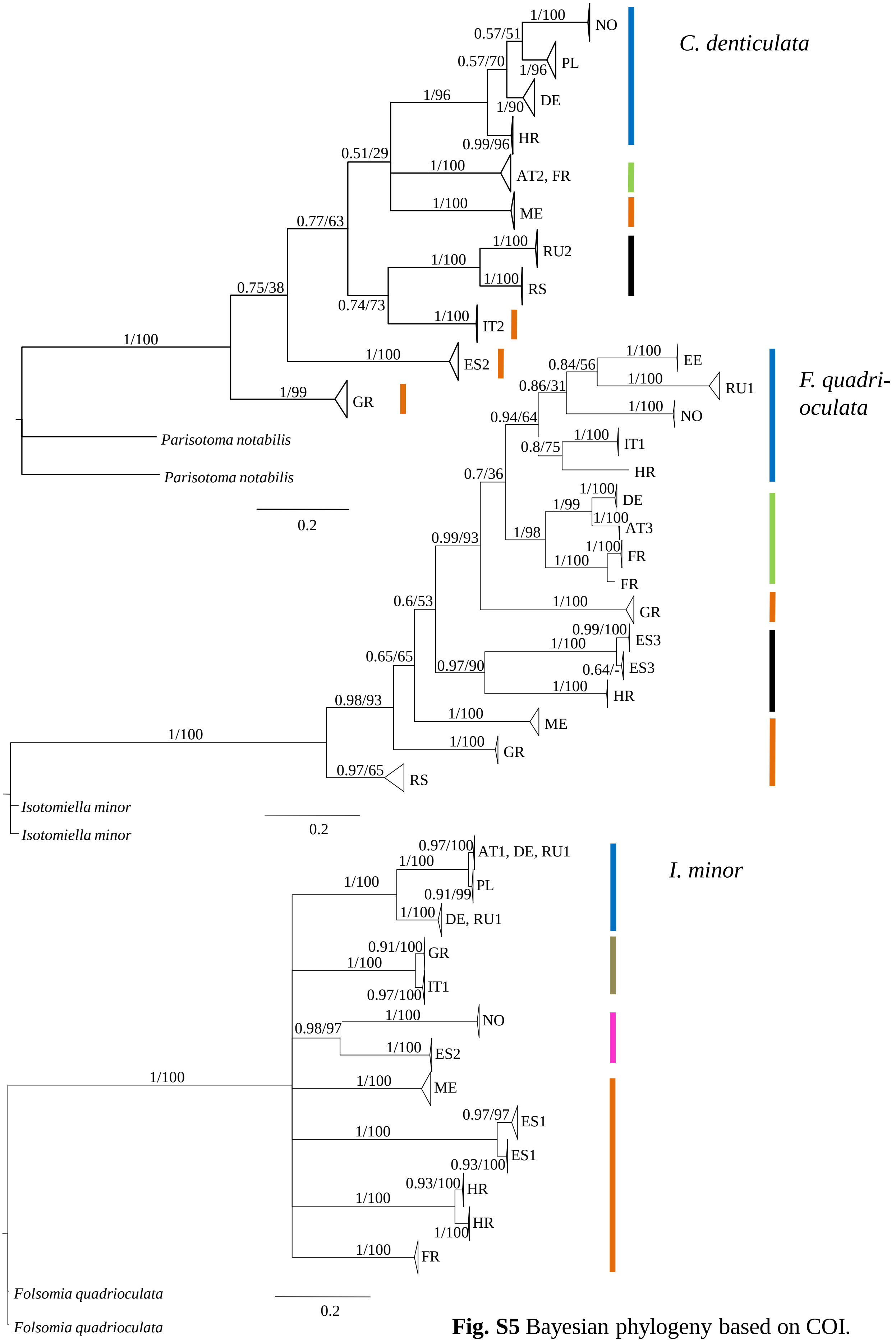

Fig. S5 Bayesian phylogeny based on COI.

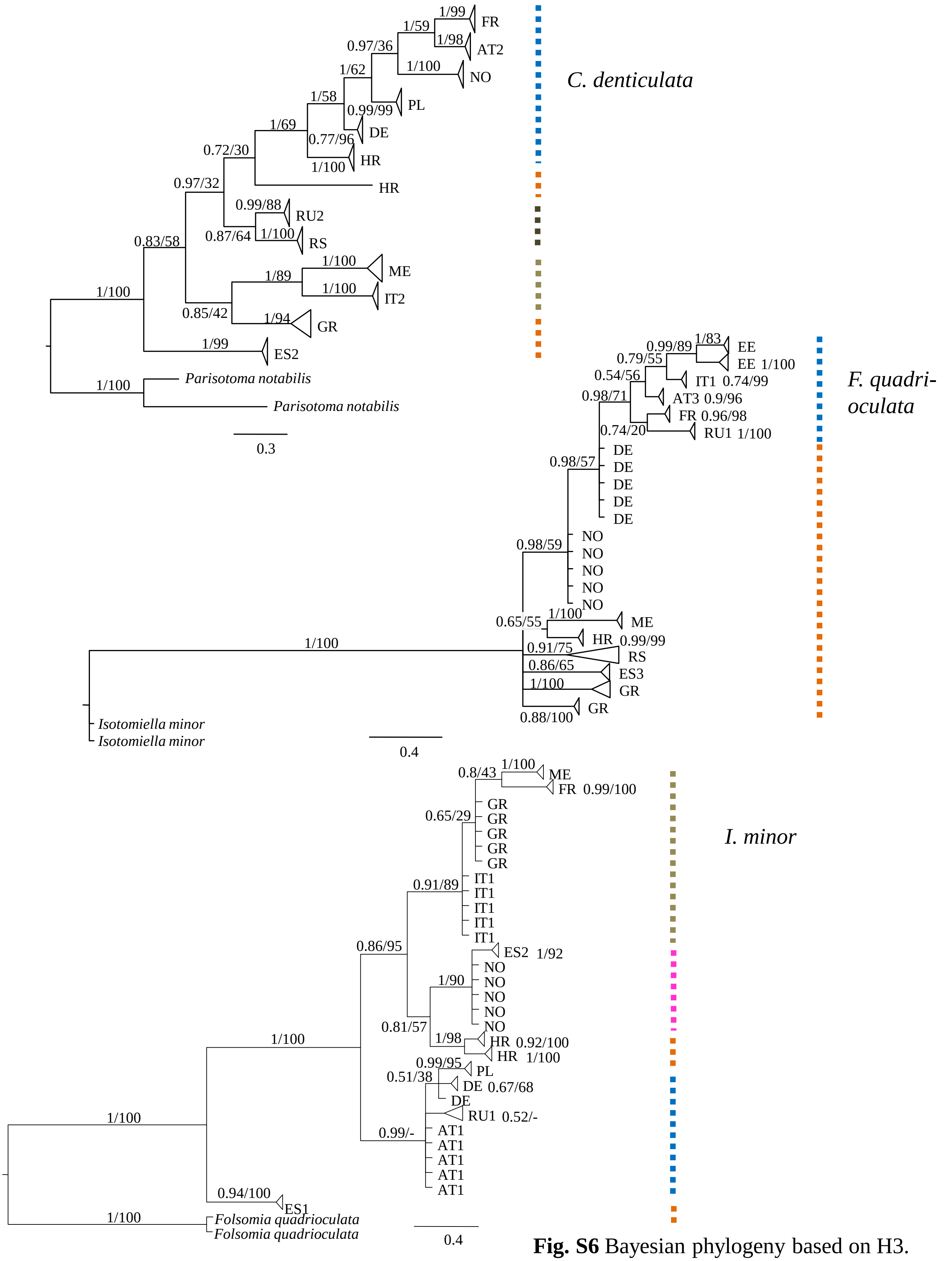

Fig. S6 Bayesian phylogeny based on H3.

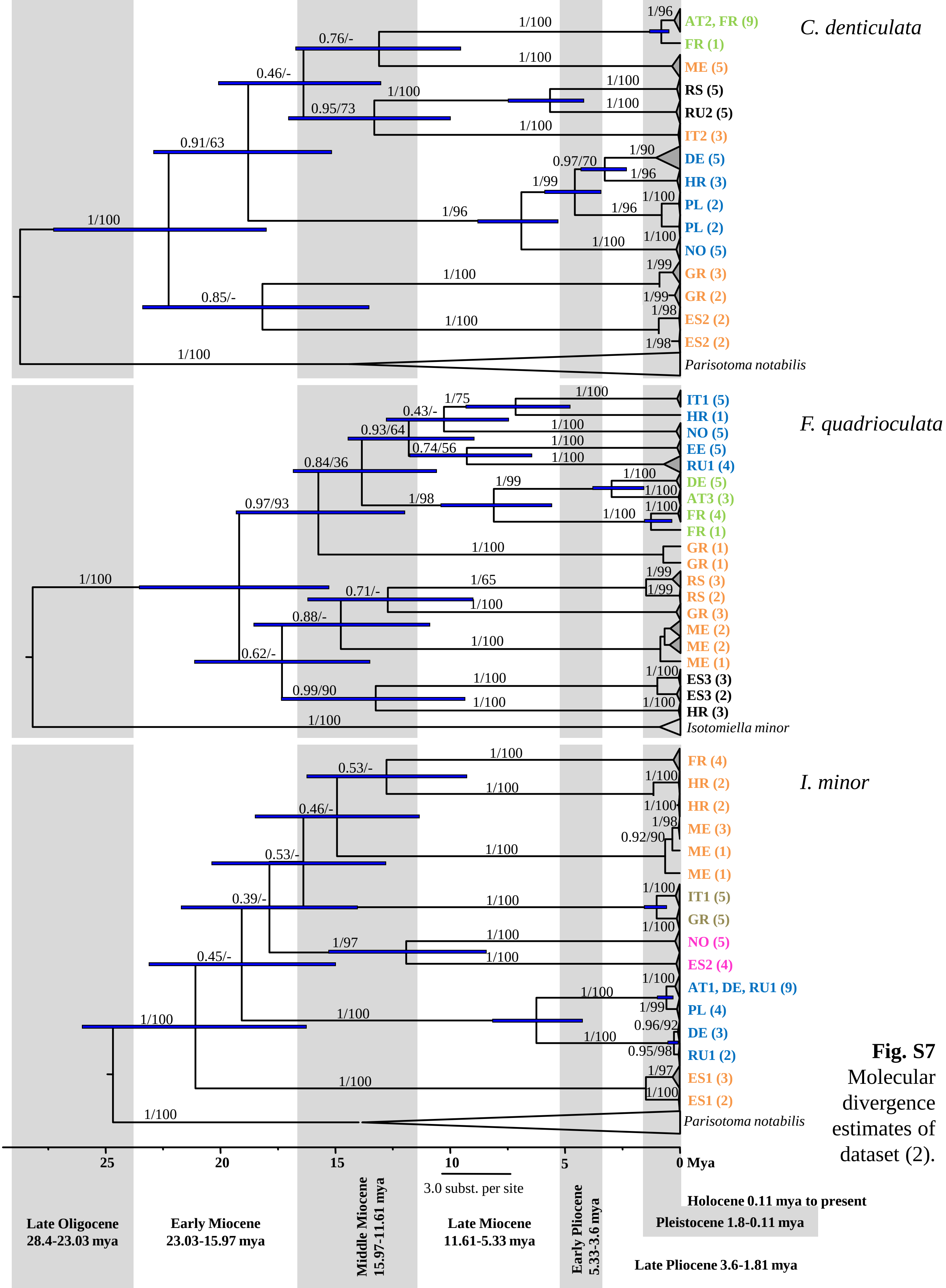

Fig. S7 Molecular divergence estimates of dataset (2).

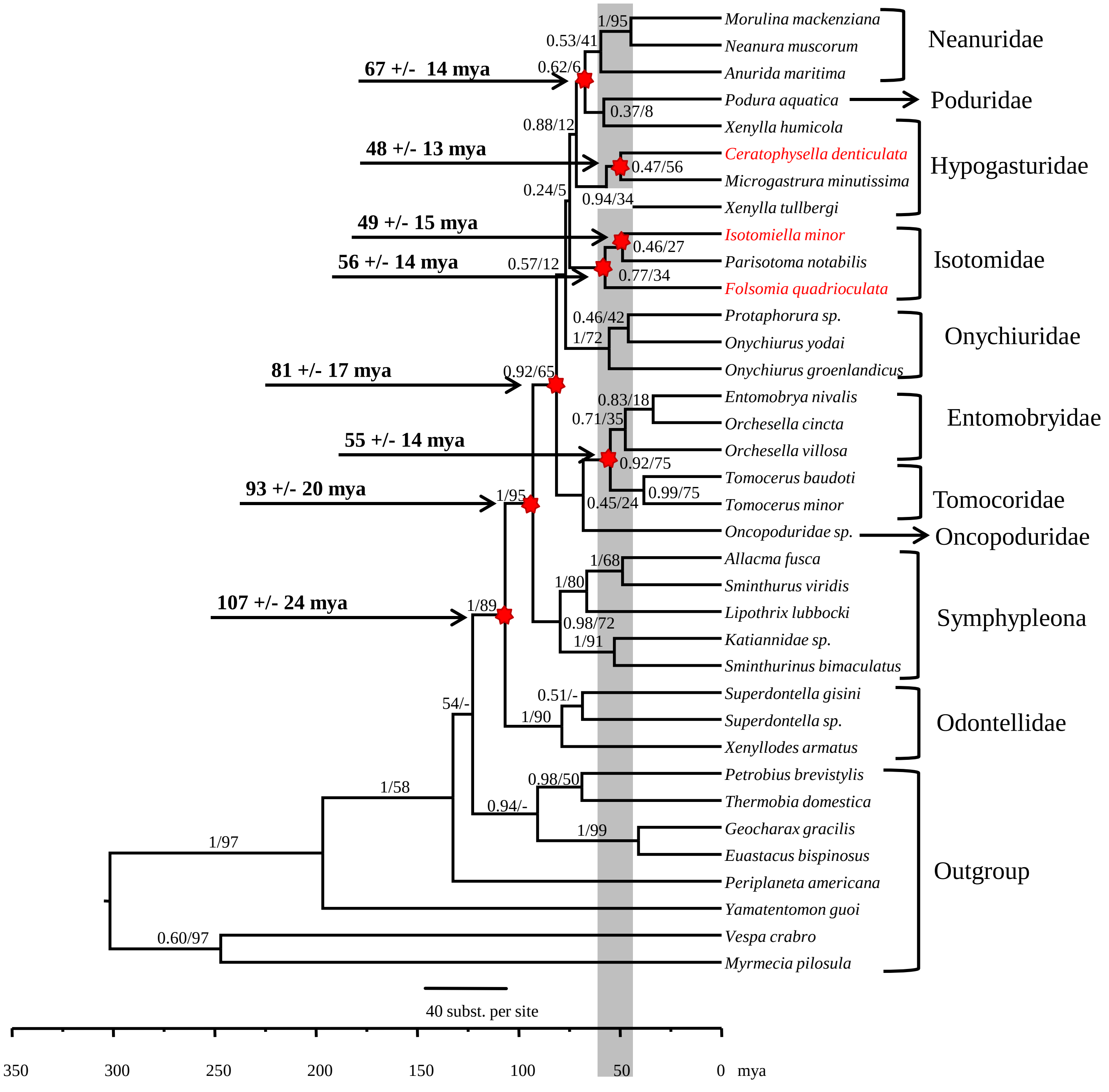

Fig. S8 Molecular phylogeny and divergence estimates of 28 species of Collembola based on COI calculated with BEAST.

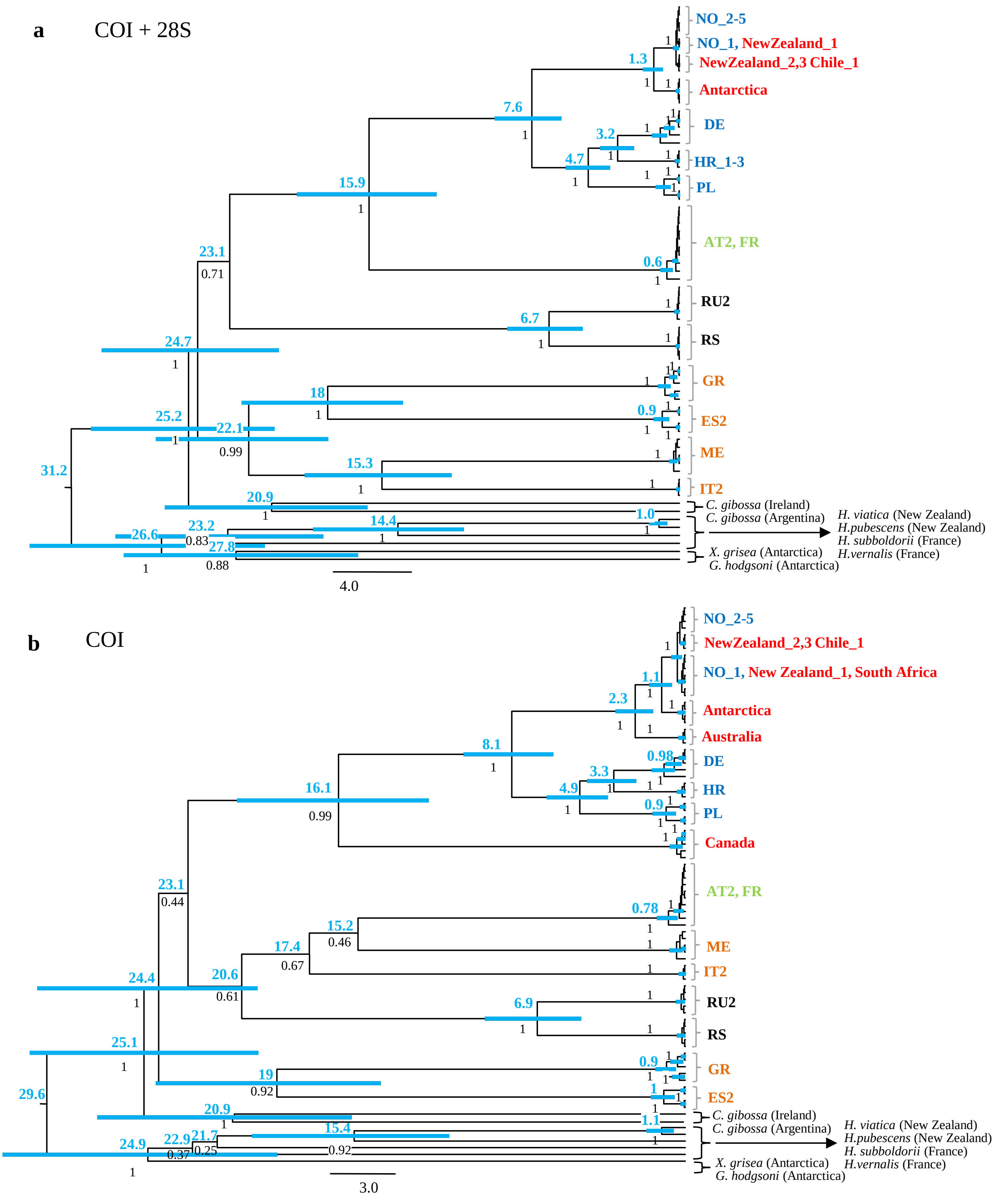

Fig. S9 Molecular divergence estimates in *C. denticulata* of a) dataset (1) and b) dataset (3). *Outgroups: C.=Ceratophysella, H.=Hypogastrura, X=Xenylla, G=Gomphiocephalus*

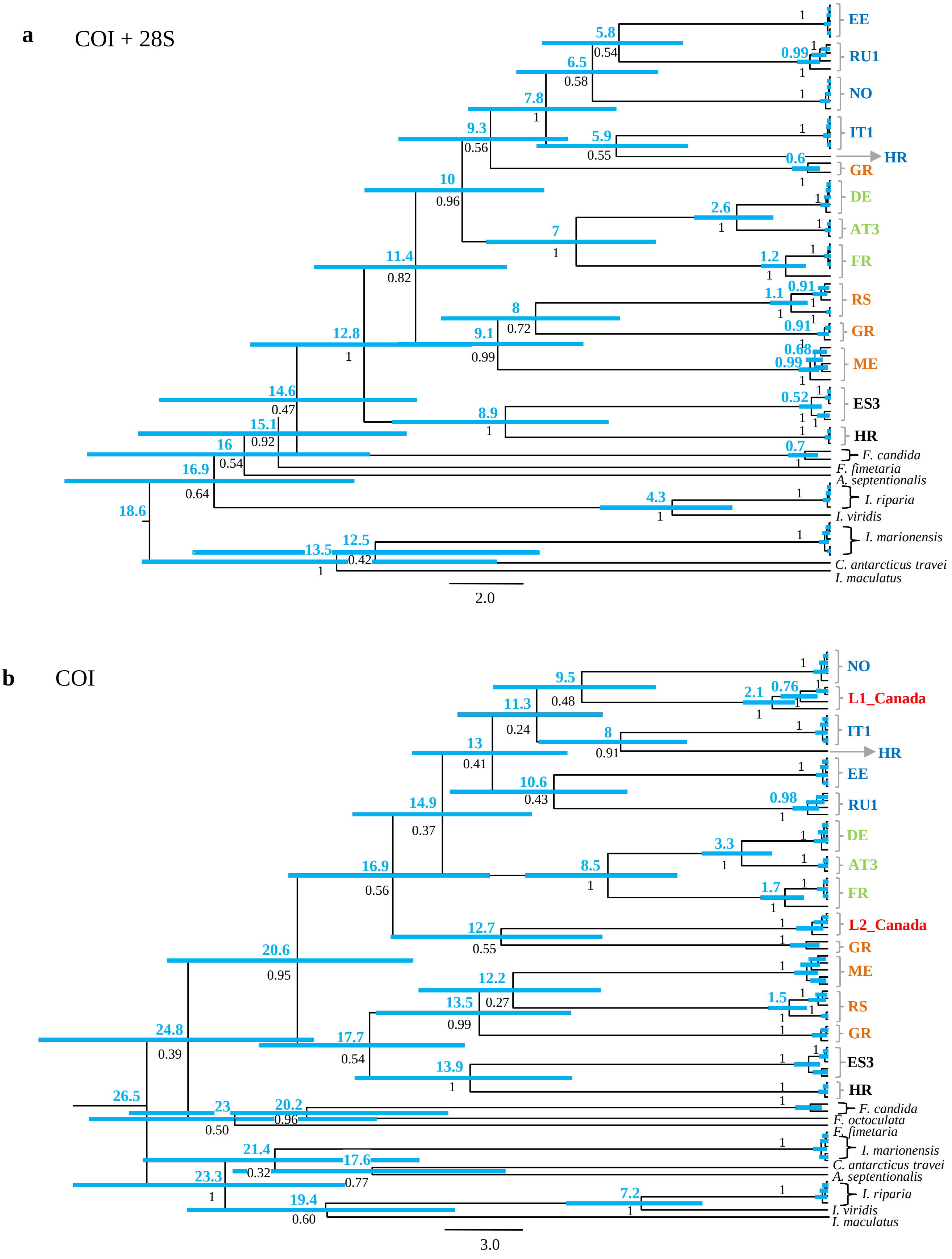

Fig. S10 Molecular divergence estimates in *F. quadrioculata* of a) dataset (1) and b) dataset (3). *Outgroups: F.=Folsomia, I.=Isotoma, C.=Cryptopygus, A.=Anurophorus*

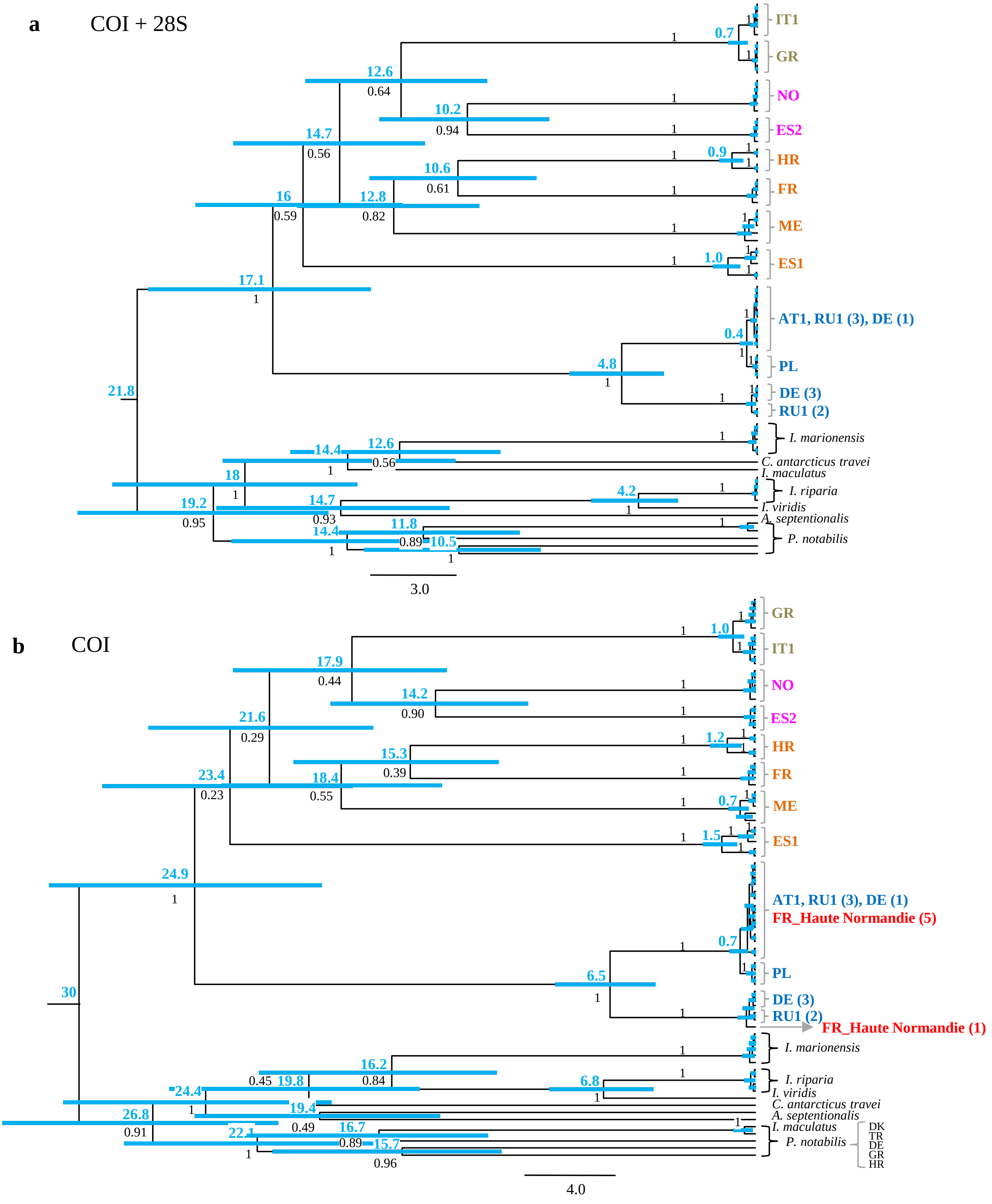

Fig. S11 Molecular divergence estimates in *I. minor* of a) dataset (1) and b) dataset (3). *Outgroups: F.=Folsomia, I.=Isotoma, C.=Cryptopygus, A.=Anurophorus*
